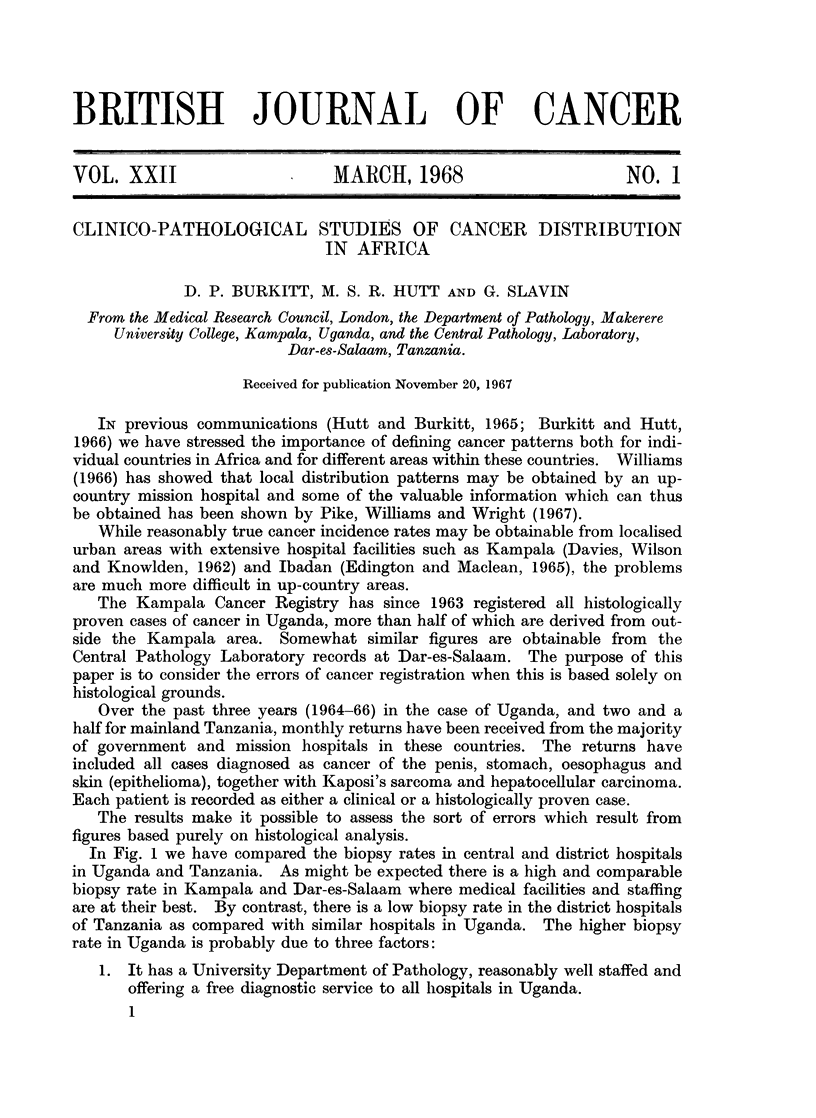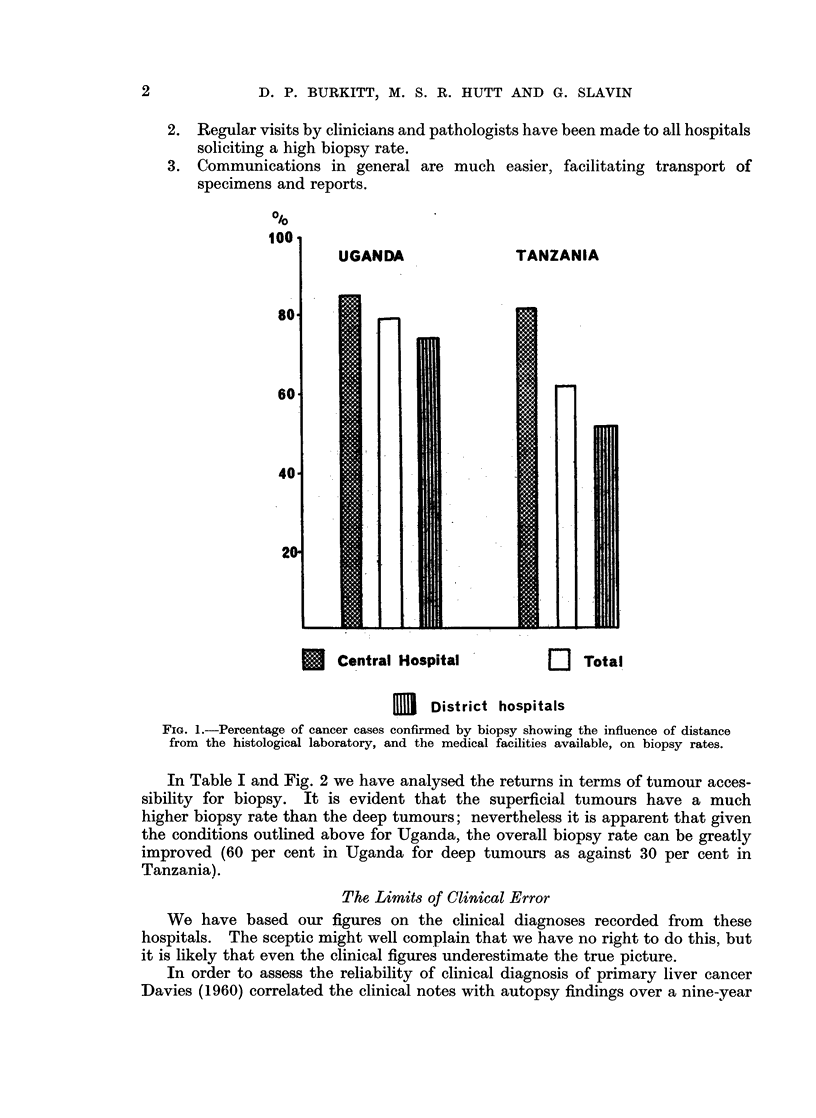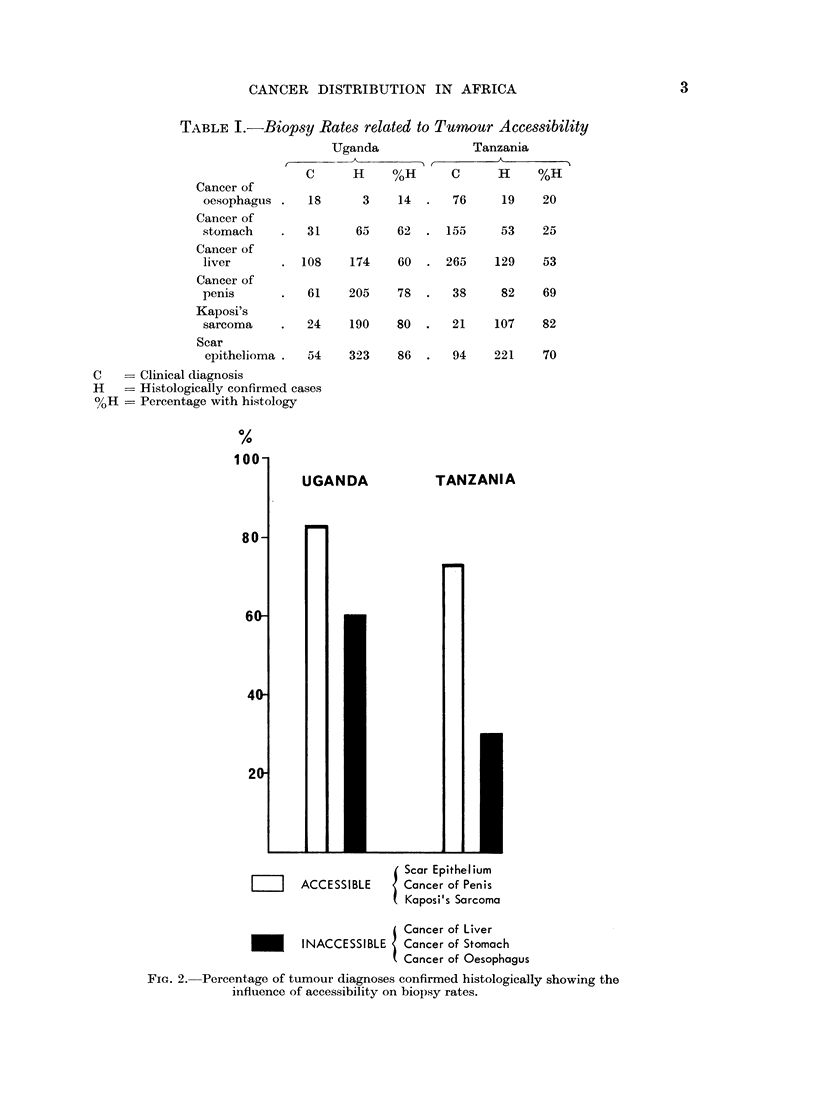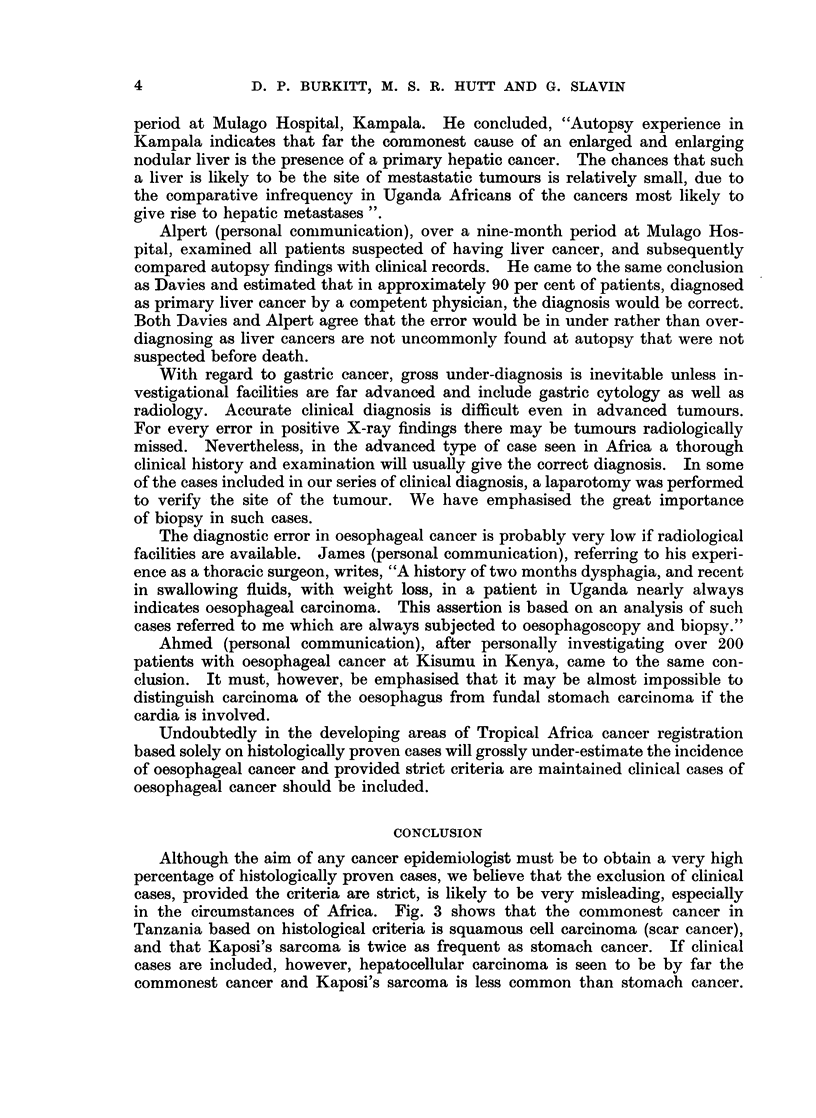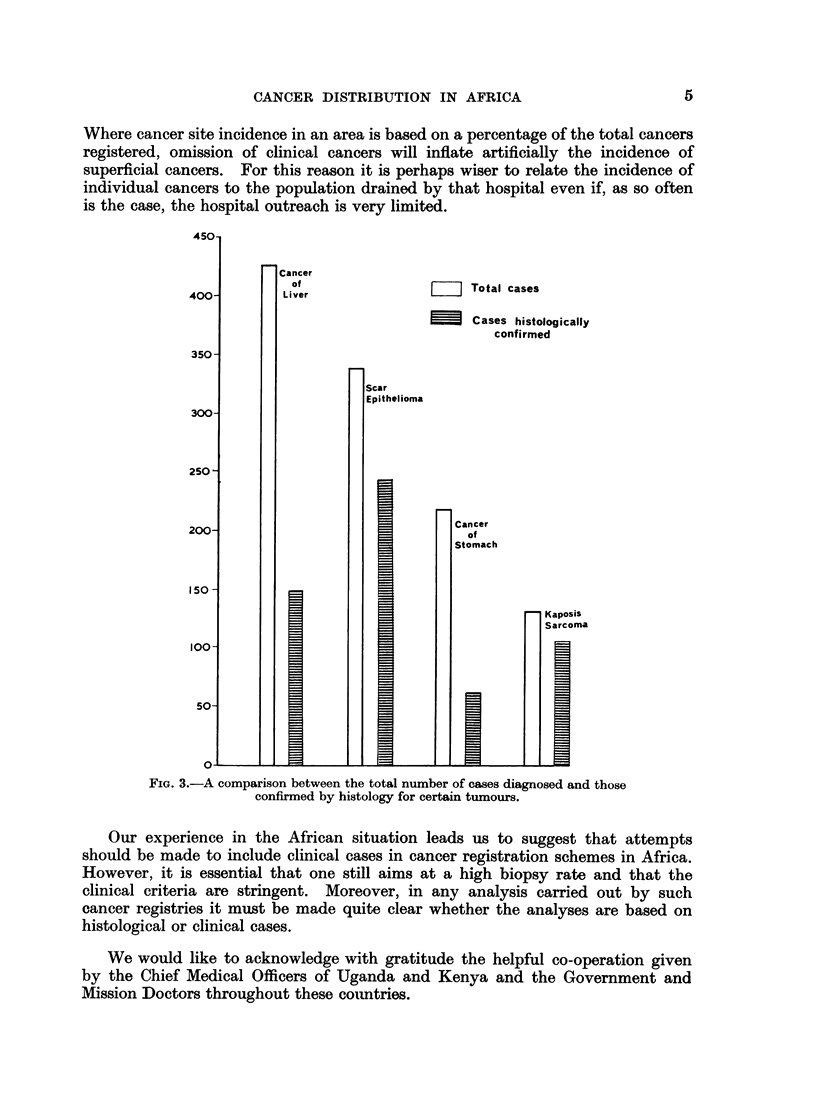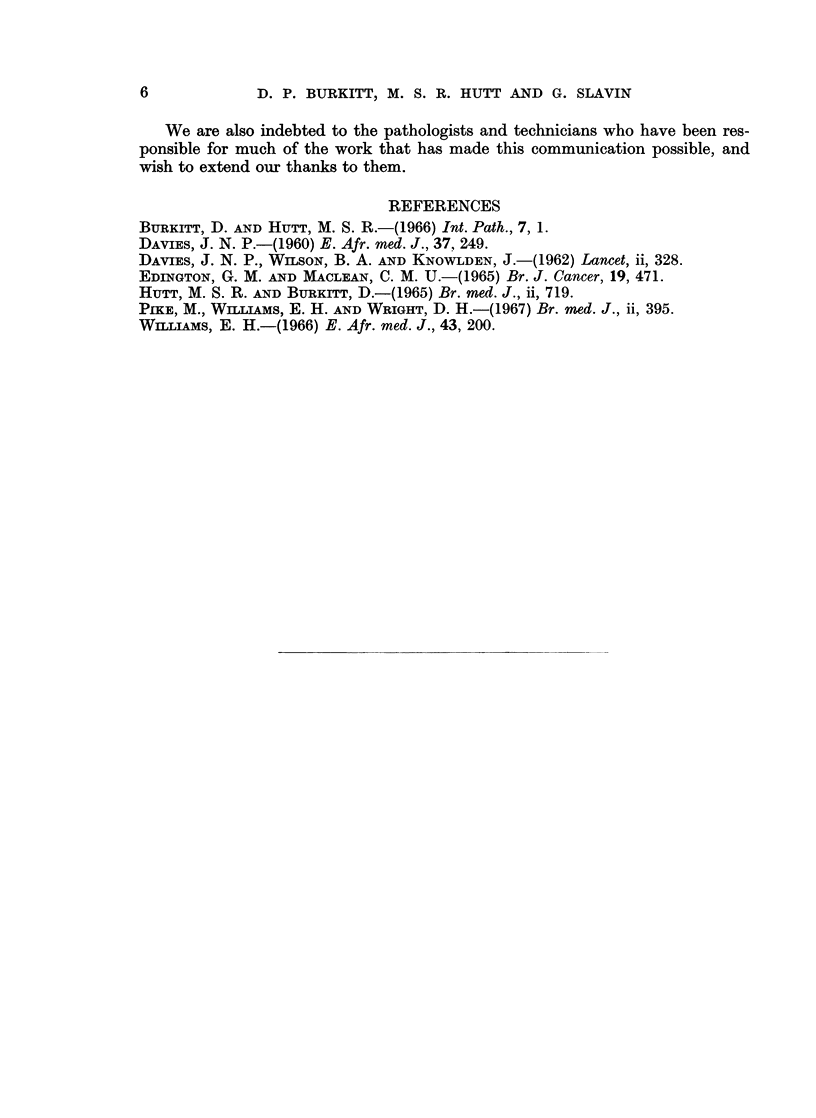# Clinico-pathological studies of cancer distribution in Africa.

**DOI:** 10.1038/bjc.1968.1

**Published:** 1968-03

**Authors:** D. P. Burkitt, M. S. Hutt, G. Slavin


### BRITISH JOURNAL OF CANCER

VOL. XXII MARCH, 1968 NO. 1

### CLINICO-PATHOLOGICAL STUDIES OF CANCER DISTRIBUTION IN AFRICA

D. P. BURKITT, M. S. R. HUTT AND G. SLAVIN

*From the Medical Research Council, London, the Department of Pathology, Makerere University College, Kampala, Uganda, and the Central Pathology, Laboratory, Dar-es-Salaam, Tanzania.*

Received for publication November 20, 1967

IN previous communications (Hutt and Burkitt, 1965; Burkitt and Hutt, 1966) we have stressed the importance of defining cancer patterns both for individual countries in Africa and for different areas within these countries. Williams (1966) has showed that local distribution patterns may be obtained by an up-country mission hospital and some of the valuable information which can thus be obtained has been shown by Pike, Williams and Wright (1967).

While reasonably true cancer incidence rates may be obtainable from localised urban areas with extensive hospital facilities such as Kampala (Davies, Wilson and Knowlden, 1962) and Ibadan (Edington and Maclean, 1965), the problems are much more difficult in up-country areas.

The Kampala Cancer Registry has since 1963 registered all histologically proven cases of cancer in Uganda, more than half of which are derived from outside the Kampala area. Somewhat similar figures are obtainable from the Central Pathology Laboratory records at Dar-es-Salaam. The purpose of this paper is to consider the errors of cancer registration when this is based solely on histological grounds.

Over the past three years (1964–66) in the case of Uganda, and two and a half for mainland Tanzania, monthly returns have been received from the majority of government and mission hospitals in these countries. The returns have included all cases diagnosed as cancer of the penis, stomach, oesophagus and skin (epithelioma), together with Kaposi's sarcoma and hepatocellular carcinoma. Each patient is recorded as either a clinical or a histologically proven case.

The results make it possible to assess the sort of errors which result from figures based purely on histological analysis.

In Fig. 1 we have compared the biopsy rates in central and district hospitals in Uganda and Tanzania. As might be expected there is a high and comparable biopsy rate in Kampala and Dar-es-Salaam where medical facilities and staffing are at their best. By contrast, there is a low biopsy rate in the district hospitals of Tanzania as compared with similar hospitals in Uganda. The higher biopsy rate in Uganda is probably due to three factors:

1. It has a University Department of Pathology, reasonably well staffed and offering a free diagnostic service to all hospitals in Uganda.

2. Regular visits by clinicians and pathologists have been made to all hospitals soliciting a high biopsy rate.
3. Communications in general are much easier, facilitating transport of specimens and reports.

FIG. 1.—Percentage of cancer cases confirmed by biopsy showing the influence of distance from the histological laboratory, and the medical facilities available, on biopsy rates.

In Table I and Fig. 2 we have analysed the returns in terms of tumour accessibility for biopsy. It is evident that the superficial tumours have a much higher biopsy rate than the deep tumours; nevertheless it is apparent that given the conditions outlined above for Uganda, the overall biopsy rate can be greatly improved (60 per cent in Uganda for deep tumours as against 30 per cent in Tanzania).

*The Limits of Clinical Error*

We have based our figures on the clinical diagnoses recorded from these hospitals. The sceptic might well complain that we have no right to do this, but it is likely that even the clinical figures underestimate the true picture.

In order to assess the reliability of clinical diagnosis of primary liver cancer Davies (1960) correlated the clinical notes with autopsy findings over a nine-year

TABLE I.—*Biopsy Rates related to Tumour Accessibility*

| | Uganda | | | Tanzania | | |
|---|---|---|---|---|---|---|
| | C | H | %H | C | H | %H |
| Cancer of oesophagus | 18 | 3 | 14 | 76 | 19 | 20 |
| Cancer of stomach | 31 | 65 | 62 | 155 | 53 | 25 |
| Cancer of liver | 108 | 174 | 60 | 265 | 129 | 53 |
| Cancer of penis | 61 | 205 | 78 | 38 | 82 | 69 |
| Kaposi's sarcoma | 24 | 190 | 80 | 21 | 107 | 82 |
| Scar epithelioma | 54 | 323 | 86 | 94 | 221 | 70 |

C = Clinical diagnosis
H = Histologically confirmed cases
%H = Percentage with histology

FIG. 2.—Percentage of tumour diagnoses confirmed histologically showing the influence of accessibility on biopsy rates.

period at Mulago Hospital, Kampala. He concluded, "Autopsy experience in Kampala indicates that far the commonest cause of an enlarged and enlarging nodular liver is the presence of a primary hepatic cancer. The chances that such a liver is likely to be the site of mestastatic tumours is relatively small, due to the comparative infrequency in Uganda Africans of the cancers most likely to give rise to hepatic metastases ".

Alpert (personal communication), over a nine-month period at Mulago Hospital, examined all patients suspected of having liver cancer, and subsequently compared autopsy findings with clinical records. He came to the same conclusion as Davies and estimated that in approximately 90 per cent of patients, diagnosed as primary liver cancer by a competent physician, the diagnosis would be correct. Both Davies and Alpert agree that the error would be in under rather than over-diagnosing as liver cancers are not uncommonly found at autopsy that were not suspected before death.

With regard to gastric cancer, gross under-diagnosis is inevitable unless investigational facilities are far advanced and include gastric cytology as well as radiology. Accurate clinical diagnosis is difficult even in advanced tumours. For every error in positive X-ray findings there may be tumours radiologically missed. Nevertheless, in the advanced type of case seen in Africa a thorough clinical history and examination will usually give the correct diagnosis. In some of the cases included in our series of clinical diagnosis, a laparotomy was performed to verify the site of the tumour. We have emphasised the great importance of biopsy in such cases.

The diagnostic error in oesophageal cancer is probably very low if radiological facilities are available. James (personal communication), referring to his experience as a thoracic surgeon, writes, "A history of two months dysphagia, and recent in swallowing fluids, with weight loss, in a patient in Uganda nearly always indicates oesophageal carcinoma. This assertion is based on an analysis of such cases referred to me which are always subjected to oesophagoscopy and biopsy."

Ahmed (personal communication), after personally investigating over 200 patients with oesophageal cancer at Kisumu in Kenya, came to the same conclusion. It must, however, be emphasised that it may be almost impossible to distinguish carcinoma of the oesophagus from fundal stomach carcinoma if the cardia is involved.

Undoubtedly in the developing areas of Tropical Africa cancer registration based solely on histologically proven cases will grossly under-estimate the incidence of oesophageal cancer and provided strict criteria are maintained clinical cases of oesophageal cancer should be included.

### CONCLUSION

Although the aim of any cancer epidemiologist must be to obtain a very high percentage of histologically proven cases, we believe that the exclusion of clinical cases, provided the criteria are strict, is likely to be very misleading, especially in the circumstances of Africa. Fig. 3 shows that the commonest cancer in Tanzania based on histological criteria is squamous cell carcinoma (scar cancer), and that Kaposi's sarcoma is twice as frequent as stomach cancer. If clinical cases are included, however, hepatocellular carcinoma is seen to be by far the commonest cancer and Kaposi's sarcoma is less common than stomach cancer.

Where cancer site incidence in an area is based on a percentage of the total cancers registered, omission of clinical cancers will inflate artificially the incidence of superficial cancers. For this reason it is perhaps wiser to relate the incidence of individual cancers to the population drained by that hospital even if, as so often is the case, the hospital outreach is very limited.

FIG. 3.—A comparison between the total number of cases diagnosed and those confirmed by histology for certain tumours.

Our experience in the African situation leads us to suggest that attempts should be made to include clinical cases in cancer registration schemes in Africa. However, it is essential that one still aims at a high biopsy rate and that the clinical criteria are stringent. Moreover, in any analysis carried out by such cancer registries it must be made quite clear whether the analyses are based on histological or clinical cases.

We would like to acknowledge with gratitude the helpful co-operation given by the Chief Medical Officers of Uganda and Kenya and the Government and Mission Doctors throughout these countries.

We are also indebted to the pathologists and technicians who have been responsible for much of the work that has made this communication possible, and wish to extend our thanks to them.

### REFERENCES

Burkitt, D. and Hutt, M. S. R.—(1966) *Int. Path.*, **7**, 1.
Davies, J. N. P.—(1960) *E. Afr. med. J.*, **37**, 249.
Davies, J. N. P., Wilson, B. A. and Knowlden, J.—(1962) *Lancet*, ii, 328.
Edington, G. M. and Maclean, C. M. U.—(1965) *Br. J. Cancer*, **19**, 471.
Hutt, M. S. R. and Burkitt, D.—(1965) *Br. med. J.*, ii, 719.
Pike, M., Williams, E. H. and Wright, D. H.—(1967) *Br. med. J.*, ii, 395.
Williams, E. H.—(1966) *E. Afr. med. J.*, **43**, 200.